# P-1360. Restoring Efficacy against Ceftaroline-Resistant MRSA with Ceftaroline-Carbapenem Combinations

**DOI:** 10.1093/ofid/ofaf695.1547

**Published:** 2026-01-11

**Authors:** Joshua A Olson, Valliammai Alaguvel, Gabriel Pérez-Parra, Anuj K Khetarpal, Valeria Rodríguez-Guevara, Vanessa Vu, George Sakoulas, Erlinda R Ulloa

**Affiliations:** UCSD/ UCI, la jolla, California; University of California, Irvine, California; University of California Irvine, Downey, California; UC Irvine School of Medicine, Irvine, California; University of California Irvine, Downey, California; Brown University, Huntington Beach, California; University of California San Diego School of Medicine, San Diego, CA; University of California Irvine School of Medicine, Irvine, California

## Abstract

**Background:**

Ceftaroline-resistant methicillin-resistant *Staphylococcus aureus* (MRSA) is an emerging threat, especially in patients with comorbidities requiring chronic antibiotic exposure. As one of the few β-lactams active against MRSA, ceftaroline's diminishing efficacy jeopardizes treatment options. Building on prior work showing ceftaroline-carbapenem synergy, we evaluated this approach against a ceftaroline-resistant strain—where its potential remains underexplored.

Carbapenems potentiate the antibacterial activity of ceftaroline against MRSA AR-0703

**Figure 1.**
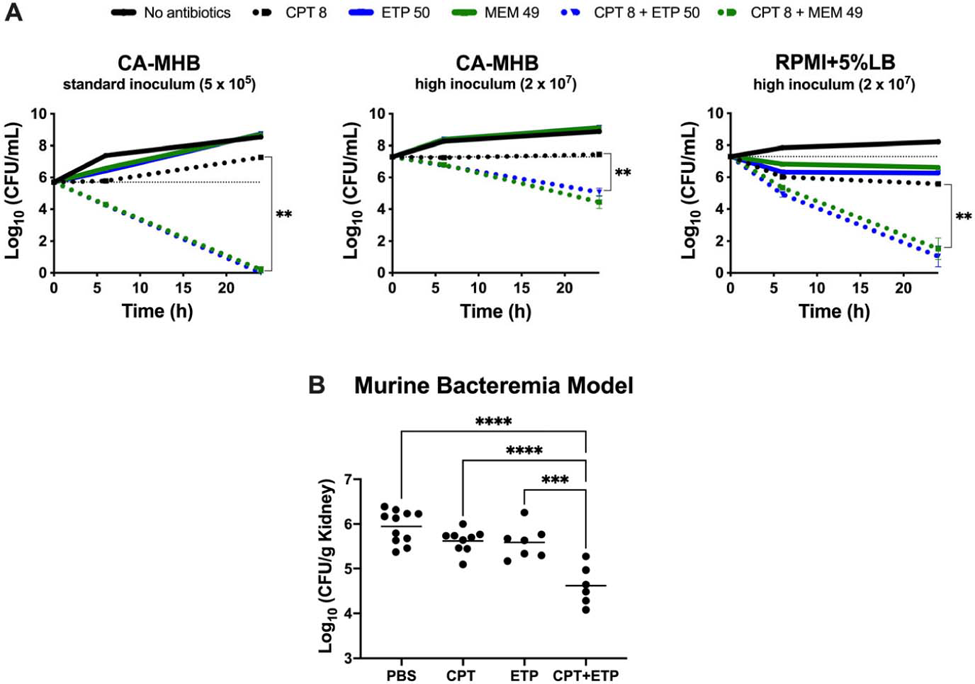
Synergistic effects of ceftaroline-carbapenem combinations against methicillin-resistant Staphylococcus aureus (MRSA AR-0703) under various conditions. (A) Kill curves over 24 hours demonstrating the effect of subtherapeutic ceftaroline (CPT, 8 mg/L, 1/2 MIC) and ertapenem (ETP, 50 mg/L) or meropenem (MEM, 49 mg/L) at average serum concentrations, alone or in combination, against ceftaroline-resistant MRSA AR-0703. Experiments were conducted under standard (5×105 CFU/mL) and high (2×107 CFU/mL) inoculum conditions in CA-MHB or RPMI+5%LB media. Combination therapy with either carbapenem showed enhanced bacterial killing compared to monotherapy, particularly under high inoculum, physiological (RPMI+5%LB) conditions. (B) Efficacy of adjunctive carbapenem therapy in a murine bacteremia model. Bacterial counts from kidneys (CFU/g) after 24 hours of treatment with ceftaroline (12 mg/kg q8 h) or ertapenem (100 mg/kg q8 h), alone or in combination, versus no antibiotics (PBS control) are shown. Combination therapy with ceftaroline (CPT) and ertapenem (ETP) significantly reduced recoverable ceftaroline-resistant MRSA from kidneys compared to both monotherapies and the PBS control (n>5). Statistical significance was determined by unpaired two-tailed t-test (A) and one-way ANOVA with multiple comparisons (B). **P ≤ 0.01, ***P ≤ 0.001, ****P ≤ 0.0001. Abbreviations: CA-MHB, cation-adjusted Mueller-Hinton broth; RPMI+5%LB, Roswell Park Memorial Institute physiological cell culture media supplemented with 5% Luria-Bertani; CFU, colony-forming units; PBS, phosphate-buffered saline.

**Methods:**

We evaluated a well-characterized ceftaroline-resistant MRSA isolate (CDC AR-0703) using checkerboard synergy testing and time-kill assays under bacteriologic (CA-MHB) and physiologic (RPMI + 5% LB) conditions. Given the association between high-inoculum staphylococcal infections—such as infective endocarditis—and clinical treatment failures, antibiotic activity was assessed at both standard (5×10⁵ CFU/mL) and high (2×10⁷ CFU/mL) inocula. *In vivo* efficacy was assessed using a murine bacteremia model. Mice received humanized, subtherapeutic regimens of ceftaroline (12 mg/kg q8h) ± ertapenem (100 mg/kg q8h), and kidney bacterial burden was quantified at 26 hours.

**Results:**

Ceftaroline combined with ertapenem or meropenem exhibited synergy by checkerboard (FICI ≤0.5) at both standard and high inocula. In time-kill assays, combinations drove marked bacterial reductions versus monotherapy, especially in physiologic media under high-inoculum conditions. *In vivo*, ceftaroline plus ertapenem reduced kidney bacterial burden by ∼1 log_10_ (p< 0.05) compared to ceftaroline alone, despite minimal activity from either agent alone.

**Conclusion:**

Ceftaroline-carbapenem combinations restored bactericidal activity against ceftaroline-resistant MRSA *in vitro* and *in vivo*, despite high MICs and limited monotherapy efficacy. These findings support their potential as a salvage strategy for serious, drug-resistant MRSA infections and warrant broader clinical investigation.

**Disclosures:**

George Sakoulas, MD, Abbvie: Honoraria

